# Supplementary material for: Correlation of Faecal Egg Counts with Clinical Parameters and Agreement between Different Raters Assessing FAMACHA©, BCS and Dag Score in Austrian Dairy Sheep
Source: Animals (Basel). 2023 Oct 13;13(20):3206. doi: 10.3390/ani13203206 (PMC10603707; doi:10.3390/ani13203206)
Supplement: Supplementary file 1 [file animals-13-03206-s001.zip › animals-2640402-supplementary.pdf]

animals

Table S1. Descriptive statistics of egg shedding in terms of eggs per gram of faeces = (EpG) originating from 1195 individual faecal samples from dairy sheep from 16 dairy sheep farms. On average 81 samples were collected per farm (range = 17 to 161 individual faecal samples).

Farm ID	N Sheep	Minimum EpG	Maximum EpG	Mean EpG	Percentiles				
					10	25	50	75	90
1	29	0.0	885.0	185.0	0.0	42.5	100.0	147.5	600.0
2	22	0.0	1120.0	186.8	10.0	18.8	70.0	270.0	695.0
3	87	30.0	12,025.0	1134.1	72.0	155.0	365.0	1515.0	3144.0
4	126	0.0	2570.0	215.4	0.0	0.0	27.5	235.0	645.5
5	95	15.0	12,035.0	1665.7	86.0	325.0	970.0	1875.0	4589.0
6	161	5.0	20,175.0	1318.0	46.0	115.0	485.0	1500.0	3212.0
7	55	0.0	1815.0	433.7	21.0	75.0	170.0	585.0	1370.0
8	22	10.0	1580.0	254.1	31.5	58.8	147.5	325.0	546.0
9	17	120.0	4615.0	1470.0	172.0	442.5	1505.0	2022.5	3151.0
10	118	0.0	3585.0	461.7	15.0	52.5	205.0	556.25	1129.5
11	119	0.0	7770.0	628.5	10.0	30.0	150.0	775.0	1910.0
12	128	0.0	6605.5	1065.9	20.0	106.3	480.0	1420.0	3375.5
13	69	0.0	5135.0	1035.9	70.0	135.0	455.0	1595.0	2980.0
14	19	0.0	2085.0	314.7	20.0	25.0	145.0	450.0	710.0
15	42	15	3100.0	352.6	23.0	50.0	177.5	376.3	1135.0
16	86	0.0	3150.0	397.2	15.0	33.8	152.5	507.5	1129.0
